# *In Ovo* Administration of Silver Nanoparticles Enhances Post-Hatch Growth Performance, Metabolic and Physiological Responses, and Immune Function in Broiler Chickens

**DOI:** 10.3390/ani16091349

**Published:** 2026-04-28

**Authors:** Hanan Al-Khalaifah, Samar A. Tolba, Inas I. Ismail, Azza A. Megahid, Eman S. Osman, Ahmed H. Rabie, Ahmed Gouda

**Affiliations:** 1 Environment and Life Sciences Research Center, Kuwait Institute for Scientific Research (KISR), Kuwait City 13109, Kuwait; hkalifa@kisr.edu.kw; 2 Department of Nutrition and Clinical Nutrition, Faculty of Veterinary Medicine, Zagazig University, Zagazig P.O. Box 44511, Egypt; 3 Poultry Breeding Department, Animal Production Research Institute, Agriculture Research Center, Giza P.O. Box 12618, Egypt; drinas53@hotmail.com (I.I.I.); drazzaabdalah@gmail.com (A.A.M.); sd_eman@yahoo.com (E.S.O.); ahmed_japan@yahoo.com (A.H.R.); 4 Department of Animal Production, National Research Center, El Buhouth St., Dokki, Giza P.O. Box 12622, Egypt; black_tiger2167@yahoo.com

**Keywords:** *in ovo* injection, silver nanoparticles, hatchability, immune response, antioxidant, broilers

## Abstract

This study examined the effects of *in ovo* silver nanoparticle (AgNP) administration on broiler chicken hatchability, growth performance, physiological and metabolic status, antioxidant capacity, and immune responses. A total of 300 fertilized Cobb500 eggs were assigned to five treatments (5 replicates/treatment, 12 eggs/replicate): non-injected control; vehicle control (0.2 mL sterile 0.9% NaCl/egg); and AgNPs at 10, 15, or 20 ppm injected into their amniotic sac on day (d) 18 of incubation. After hatching, 48 chicks/treatment were assigned to 6 replicate cages (8 birds/replicate) for 35 d. *In ovo* AgNP administration improved hatchability and reduced embryonic mortality (*p* < 0.05). Post-hatch, birds originating from AgNP-injected eggs had an improved body weight, body weight gain, and feed conversion ratio, with lower feed intake (*p* < 0.05). AgNP administration elevated hemoglobin, packed cell volume, growth hormone, serum proteins, alanine aminotransferase activity, and creatinine levels (*p* < 0.05). AgNPs also improved the lipid profile and increased total antioxidant capacity, catalase and superoxide dismutase activities, lysozyme activity, complement 3, and cytokine concentrations and reduced malondialdehyde levels (*p* < 0.05). Effects were dose-dependent, with 15 ppm generally leading to better outcomes. These findings suggest that 15 ppm AgNP *in ovo* administration enhances embryonic viability, physiological resilience, and post-hatch performance in broiler chickens.

## Simple Summary

Under modern commercial poultry production and intensive farming conditions, birds are often subjected to physiological challenges that can disrupt immune function and metabolic status, highlighting the necessity for strategies to enhance their nutritional and health status. The *in ovo* feeding strategy offers an effective method to improve embryonic development and post-hatch performance in broiler chickens. This study investigated whether supplementing silver nanoparticles (AgNPs) into chicken embryos could improve incubation outcomes and post-hatch performance in a dose-dependent manner, with an optimal level-enhancing physiological and metabolic status and immune function without adverse impacts. AgNPs were selected due to their potential to influence intestinal health, immune functions, and nutrient utilization. Fertilized eggs were injected with different AgNP doses, and birds were monitored post-hatch. The results show that AgNPs enhanced hatchability and reduced embryonic mortality while enhancing growth performance. AgNPs also had positive effects on metabolic status, antioxidant status, and immune responses, suggesting improved physiological resilience. These effects were dose-dependent, with 15 ppm of AgNPs generally showing better responses. Overall, administrating appropriate levels of AgNPs *in ovo* may represent a potential nutritional strategy to improve metabolic status, immune competence, and productivity of broiler chickens in modern production conditions.

## 1. Introduction

Poultry production is a fundamental subsector of global agriculture and serves as a significant supply of high-quality animal protein globally. This industry is characterized by the rapid integration of advanced technologies aimed at improving production efficiency. Among the primary objectives of poultry production are the optimization of the incubation process and the improvement of post-hatch growth performance, as early-life conditions profoundly influence long-term broiler productivity [1,2]. During the early post-hatch period, newly hatched chicks undergo a critical metabolic and physiological transition from yolk-based nutrition to exogenous feed intake [3,4]. Under modern commercial poultry production conditions, hatchlings are frequently exposed to prolonged fasting periods of 24–36 h (h) before access to feed and water [5]. This delay necessitates the mobilization of residual yolk proteins and body reserves to meet immediate energy demands, potentially compromising early growth and immune function [6,7]. As a result, considerable research efforts have gone into enhancing the nutritional status of embryos through *in ovo* supplementation. This method has been shown to improve post-hatch growth performance, development of the gastrointestinal tract, and immune competence, while also reducing the dependence of embryonic development on the nutritional status of the breeder hen [8,9,10,11,12].

In recent years, nanotechnology, which entails the manipulation of materials at dimensions ranging from 1 to 100 nanometers, has acquired significant traction in agricultural and animal sciences because of its distinctive structural and functional features [13]. In the context of poultry nutrition, nanoscale treatments present innovative approaches to address the shortcomings of conventional feed additives [14,15]. Their diminutive particle size and extensive surface area augment the solubility, absorption, and stability of nutrients and bioactive compounds within the gastrointestinal tract [15]. Among various nanoparticles, silver nanoparticles (AgNPs) have received considerable interest due to their potent antibacterial and immunomodulatory capabilities [16,17]. AgNPs exhibit broad-spectrum biocidal activity, particularly against Gram-negative bacteria, which has driven their increasing incorporation into a wide range of biomedical and medicinal products [18,19]. In poultry production, AgNPs are proposed to influence intestinal microbial communities and promote the overall health and immunological status of birds, thereby allowing a greater proportion of nutrients to be redirected into growth and other physiological and productive functions [20,21]. However, despite these promising attributes, further research is required to elucidate the long-term effects of *in ovo* AgNP supplementation and to fully assess its potential benefits and safety for the poultry production industry.

Previous studies have demonstrated that *in ovo* administration of AgNPs can enhance immune response in poultry; specifically, Goel et al. [17] indicated that *in ovo* administration of AgNPs at 15 µg/egg augmented in vivo immune responses to phytohemagglutinin-p and sheep red blood cells. Similarly, Beck et al. [22] indicated that *in ovo* administration of AgNPs at a level of 50 mg/L, whether administered alone or in combination with hydroxyproline, enhances embryonic development, post-hatch performance, and immunological function in poultry. Mechanistic evidence further suggests that *in ovo* administration of 20 ppm of AgNPs has been linked to the elevated expression of fibroblast growth factor 2 and vascular endothelial growth factor, which are essential regulators of satellite cell proliferation and differentiation of broilers [23]. Recently, Mahini and colleagues revealed that *in ovo* AgNP administration influenced productive characteristics and hepatic gene expression in broilers subjected to a liposaccharide challenge, suggesting that early AgNP exposure may impact subsequent physiological responsiveness to stress later in life [24]. Nevertheless, despite these earlier findings, limited information is available regarding the dose–response relationship of *in ovo*-administered AgNPs, particularly in studies that integrate post-hatch growth performance with comprehensive systemic physiological responses.

Therefore, the present study was designed to evaluate the effects of *in ovo* administration of AgNPs at different concentrations on hatchability and growth performance of broiler chickens. In addition, the study aimed at investigating the associated changes in blood biochemical parameters, antioxidant status, and immune response markers during the grow-out period. We hypothesized that *in ovo* administration of AgNPs would improve incubation outcomes and post-hatch performance in a dose-dependent manner, with an optimal dose-enhancing physiological status without adverse impacts.

## 2. Materials and Methods

The experiment herein was executed by the Department of Animal Production at the National Research Center, Dokki, and the Department of Nutrition and Clinical Nutrition, Faculty of Veterinary Medicine, Zagazig University. The Animal Protocol received approval from the Institutional Animal Care and Use Committee at Zagazig University (ZU-IACUC; approval no: ZU-IACUC/2/F/284/2026).

### 2.1. Nanoparticles

Commercial AgNPs were procured from Fortis Life Science, Inc. (NanoComosix^®^, San Diego, CA, USA), as a citrate-stabilized colloidal suspension. According to the manufacturer’s certificate of analysis, the AgNPs had a spherical morphology with an average primary particle diameter of 20 ± 3 nm and were supplied at a stock concentration of 1 mg/mL. Particle size distribution and colloidal stability were verified by the manufacturer using transmission electron microscopy and dynamic light scattering. Prior to *in ovo* administration, the AgNP stock suspension was diluted with a sterile 0.9% sodium chloride solution to prepare working concentrations of 10, 15, and 20 ppm, in accordance with the experimental design. Each egg in the AgNP treatments received 0.2 mL of the prepared nanoparticle suspension. All nanoparticle suspensions were freshly prepared on the day of injection [22].

### 2.2. Experimental Design and in Ovo Feeding

A total of 300 fertilized eggs (average weight: 63 ± 2.2 g) were obtained from a commercial hatchery (Dakahlia Poultry, Mansoura, Egypt). All eggs were obtained from the same Cobb500 breeder flock (46 weeks of age) and were collected within 24 h (h) of laying. Eggs were stored at 14–16 °C for 24 h prior to incubation. All eggs were placed into incubator trays and randomly assigned to five experimental treatments (60 eggs/group; 5 replicates/treatment, 12 eggs/replicate). The first group served as a negative control (NC) and received no injection. The second group served as the vehicle control (VC) and received an injection of 0.2 mL of a sterile 0.9% sodium chloride solution per egg. The third, fourth, and fifth groups received injections of 0.2 mL per egg of AgNP suspensions at a concentration of 10, 15, and 20 ppm and were designated as AgNP10, AgNP15, and AgNP20, respectively, with the injection volume kept constant throughout all injected groups. All eggs were incubated under standard conditions (37.8 °C and 60% relative humidity (RH)) in an automatic incubator (Zhicheng Incubation Equipment Co., Ltd., Dezhou, China). On the 18th day (d) of incubation, candling was performed to confirm embryo viability prior to *in ovo* injection. At this stage, non-viable embryos were identified and excluded from further procedures [25,26,27].

The *in ovo* injection was performed under a laminar flow system, following the procedure of Ohta and Kidd [28]. Injections were directed into the air cell of the egg through a shell opening using a 21-gauge needle, subsequent to disinfection with a 70% ethanol solution. Following the injection, the shell opening was sealed with parafilm tape (Sigma-Aldrich, St. Louis, MO, USA), and eggs were transferred for continued incubation. During the hatching period, incubation conditions were adjusted to 37.2 °C and 70% RH, and the eggs were transferred from the setter trays to the hatcher baskets [27].

### 2.3. Hatchability and Embryonic Mortality

Upon hatching, the number of hatched chicks and unhatched eggs were recorded to determine hatchability and embryonic mortality percentages (%) according to Fulla & Gebreslassie [29], as follows:

Hatchability (%) = (Number of hatched chicks/Total number of fertile incubated eggs) × 100 (1)

Embryonic mortality (%) = (Number of dead embryos/Total number of fertile incubated eggs) × 100 (2)

### 2.4. Post-Hatch Housing and Management

Upon hatching, chicks remained in the incubator under their respective treatments until all were taken out for grow-out and were deemed viable when completely hatched, mobile and possessing dry-down [30]. Chicks were allocated to their respective experimental treatment groups. A total of 48 chicks per treatment were randomly selected, weighed, and assigned to 6 replicate cages per treatment, with 8 birds in each replicate. Chicks were accommodated in a cage system divided by wire mesh barriers (1.0 m × 0.50 m × 0.40 m) under consistent sanitary and managerial settings over the experimental duration (35 d). During the early post-hatch period, the room temperature was maintained at 31 °C, with a lighting schedule of 24 h of continuous light on the day of placement to promote sufficient feed and water consumption and to encourage chick activity. From day 2 post-hatch, a lighting schedule of 23 h of light and 1 h of darkness (23L:1D) was implemented. The room temperature was sustained at 31 °C till d 6, went down to 29 °C on d 7, and thereafter declined to 26 °C until d 14. Subsequently, the temperature was decreased by 1 °C every 2 d until it reached 22 °C, which was sustained until the end of the experiment.

Beginning on d 5, a lighting schedule of 18L:6D was implemented until d 20, followed by 19L:5D from d 21 to d 27, and 20L:4D from d 28 to d 35. All management measures, including lighting and temperature programs and general husbandry, were carried out in accordance with the Cobb500 Broiler Management Guide [31]. Throughout the experiment, fresh water and corn–soybean meal-based feed in mash form were provided *ad libitum*. The birds were fed the starter diet until they were 12 d of age, followed by a grower 1 and grower 2 diet from d 13–28 and d 29–35, respectively. All diets were designed to fulfill the nutrient requirements of broiler nutrient specifications of Cobb500 broilers [32] (Table 1).

### 2.5. Growth Performance

The individual body weight (BW) for all chicks was recorded at hatch. Subsequent to post-hatch distribution of experimental treatment groups, chicks were weighed at placement, and those weights were considered the initial BW (W_1_). The final BW (W_2_) was recorded at 35 d of age. Body weight gain (BWG) was determined for the entire experimental duration (1–35 d) as BWG (g/bird) = W_2_ − W_1_. Feed intake (FI) was calculated for each pen over the entire experimental period as FI (g/bird) = (feed offered − feed residuals)/number of birds. The feed conversion ratio (FCR) was then determined for the 1–35 d period as FCR = FI (g)/BWG (g) [33].

### 2.6. Sample Collection, Rectal Temperature, and Hematological Analyses

At 35 d post-hatch, all birds underwent an acute heat challenge (~40.5 °C) for 1 h before sampling to elicit a cellular-stress response and enhance heat shock protein 70 (HSP70) expression. Immediately after heat exposure, the rectal temperature (RT) was recorded, followed by collection of blood and tissue samples. The rectal temperature was measured using a digital thermometer (HANNA instruments Inc., Padova, Italy) by inserting the probe into the cloaca until the temperature readings stabilized [34]. Subsequently, six birds per treatment were randomly selected for blood and tissue sampling. For hemoglobin (Hb) and packed cell volume (PCV) analysis, approximately 1 mL of blood was collected via wing vein puncture from each bird using heparinized syringes and transferred to anticoagulant-containing tubes, then promptly placed on ice in a cooled container until analysis. Hemoglobin concentration was measured using the cyanmethemoglobin method as outlined by Drabkin and Austin [35], and PCV was determined using the microhematocrit method according to Thrall and Weiser [36]. For serum biochemical analysis, additional blood samples were obtained from the same birds, placed into anticoagulant-free vacutainer tubes (BD Vacutainer^®^, Becton Dickinson, Franklin Lakes, NJ, USA), and allowed to clot at room temperature. Samples were then centrifuged for 13 min at 3000× *g*, after which the serum was collected into Eppendorf tubes and stored at −20 °C until further examination. Following blood collection, the same birds were euthanized via cervical dislocation and eviscerated, and liver samples were dissected out, vacuum-sealed, and stored at −20 °C for subsequent HSP70 analysis.

### 2.7. Serum Biochemical Analysis

Serum total protein (TP) concentration was assessed using the biuret method, in which proteins react with copper sulfate in an alkaline sodium hydroxide solution to form a violet-colored biuret complex. Absorbance was measured spectrophotometrically at 546 nm, and color intensity was directly proportional to the protein concentration [37,38]. Serum albumin concentration was assessed using a bromocresol green (BCG) dye-binding method, based on the formation of a stable albumin–BCG complex. The resulting absorbance was measured at 620 nm [39,40]. Serum globulin concentrations were calculated by subtracting albumin from TP. The activities of alanine aminotransferase (ALT) and aspartate aminotransferase (AST) were assessed utilizing the ALT and AST activity assay kit (ALT: Cat No. ab241035; AST: Cat No. ab105135; Abcam, Waltham, MA, USA) in accordance with the manufacturer’s protocol. Serum glucose concentration was measured using a glucose colorimetric assay kit (Cat No. 10009582, Cayman Chemical, Ann Arbor, MI, USA) following the manufacturer’s instructions. Likewise, kidney function tests in terms of serum creatinine and urea levels were determined using commercial assay kits (Creatinine: Cat No. ab65340; Urea: Cat No. ab83362; Abcam, Waltham, MA, USA) according to the manufacturer’s guidelines. Growth hormone (GH) concentration was measured using a GH ELISA Kit (Cat No. MBS703063, MyBioSource, Inc., San Diego, CA, USA) following the manufacturer’s protocol.

### 2.8. Lipid Profile Analysis

Serum total cholesterol (TC), triglycerides (TG), and high-density lipoprotein cholesterol (HDL-C) concentrations were assessed utilizing commercial colorimetric diagnostic kits (Specterum Diagnostics, an Egyptian company for Biotechnology, Cairo, Egypt) according to the methods of Allain et al. [41] for TC, McGowan et al. [42] for TG, and Vassault et al. [43] for HDL-C. The serum low-density lipoprotein cholesterol (LDL-C) level was calculated using the Friedewald equation: LDL-C = TC − (HDL-C) − (TG/5) [44]. Serum very low-density lipoprotein cholesterol (VLDL-C) concentration was estimated as VLDL-C = TG/5 [44,45]. All lipid concentrations are expressed in mg/dL.

### 2.9. Antioxidant Status Assessments

Serum total antioxidant capacity (TAC) was assessed calorimetrically using commercial assay kits (Rel Assay Diagnostics, Sehitkamil/Gaziantep, Turkey) based on the method described by Janaszewska and Bartosz [46]. In this assay, antioxidants present in the serum reduce the dark blue-green 2,2′-azinobis (3-ethylbenzothiazoline-6-sulfonic acid) diammonium salt (ABTS) radical action to a colorless form. The resulting decrease in absorbance, measured at 660 nm, is proportional to the TAC of the sample. Serum superoxide dismutase (SOD) and catalase (CAT) activities were determined according to the methods established by Kraljević et al. [47]. SOD activity was measured based on the generation of superoxide radicals produced by the reaction between xanthine and xanthine oxidase, which subsequently reduce nitro blue tetrazolium (NBT) to form a colored formazan product. The rate of formazan formation is proportional to superoxide production and is inhibited in the presence of SOD. Absorbance was measured at 560 nm, and SOD activity was calculated from the degree of inhibition. Catalase activity was determined by monitoring the decomposition of hydrogen peroxide (H_2_O_2_) spectrophotometrically at 240 nm. Catalase activity was expressed as the amount of enzyme necessary to degrade 1 mmol of H_2_O_2_ per minute at pH 7.8 and 25 °C. Malondialdehyde (MDA) concentrations were determined using the thiobarbituric acid reactive substance (TBARS) assay according to the method of McDonald and Hultin [48], employing 2-thiobarbituric acid as the reacting reagent and 1,1,3,3-tetraethoxypropane as the calibration standard.

Liver tissue samples (~1 gm per sample) were rinsed three times with a cold buffer to remove residual blood and subsequently homogenized in 4 mL of a protein extraction buffer using a Polytron Homogenizer (Heat System UltraSonics, New York, NY, USA). The homogenates underwent centrifugation at 3000× *g* for 15 min at 4 °C, and the resulting supernatants were retrieved for TP determination. The HSP70 concentration was measured in triplicate using a commercial enzyme-linked immunosorbent assay kit (Quantikine, R&D Systems, Minneapolis, MN, USA) following the manufacturer’s guidelines. HSP70 values were normalized to the TP concentration and expressed as HSP70 per unit of TP.

### 2.10. Immune Status Evaluation

Serum concentrations of interleukin-10 (IL10) and complement 3 (C3) were determined using chicken-specific ELISA kits (IL10: Cat. No. MBS701683, MyBioSource Inc., San Diego, CA, USA; C3: Cat. No. LS-F9287, LifeSpan Biosciences Inc., Seattle, WA, USA) according to the manufacturers’ instructions. Serum lysozyme activity was determined using the turbidimetric method described by Lie et al. [49]. Pro-inflammatory cytokines, including interferon-gamma (IFN-γ) and interleukin-1 beta (IL-1β), were measured using chicken-specific ELISA kits (IFN-γ: Cat. No. MBS2020832; IL-1β: Cat. No. MBS2024496; MyBioSource Inc., San Diego, CA, USA) following the manufacturer’s protocols.

### 2.11. Statistical Analysis

Data were analyzed using the SPSS software (Version 28.0.1.0 for macOS 10.15, Chicago, IL, USA). Treatment effects were evaluated using one-way analysis of variance (ANOVA), and significance was determined using the F-test. Linear and quadratic dose–response effects of *in ovo* AgNP administration were tested using polynomial orthogonal contrasts. The replicate pen was considered the experimental unit for growth performance parameters, whereas the individual bird served as the experimental unit for blood biochemical, antioxidant, and immunological measurements. When significant treatment effects were detected, means were separated using Tukey’s honestly significant difference test. Results are presented as means with the pooled standard error of the means (SEM). Differences were considered statistically significant at *p* < 0.05.

## 3. Results

### 3.1. Hatchability and Growth Performance

The effects of *in ovo* AgNP injection on hatchability percentage, embryonic mortalities, and growth performance of broiler chickens are summarized in Table 2. Hatchability, calculated from set eggs, increased in AgNP-injected groups (*p* < 0.001). Importantly, Hatchability, calculated from AgNP-injected eggs at d 18, which reflect post-injection survival, also increased relative to the VC and NC (*p* < 0.001), with detected linear (*p* < 0.001) increases. Embryonic mortality (post-injection) decreased in all AgNP *in ovo* injected groups relative to the controls (*p* < 0.001), showing a linear response (*p* < 0.001). Chicks originating from eggs injected with 15 ppm of AgNPs had a higher hatch BW compared to the controls (*p* = 0.002), with a linear effect observed (*p* < 0.001). However, the initial BW at placement was not different among treatments (*p* > 0.05).

At 35 d of age, BW and BWG were higher in chickens originating from AgNP-injected eggs relative to the controls, with the highest values recorded in AgNP15 (*p* < 0.001). Both indices exhibited linear responses to increasing AgNP levels (*p* < 0.001). Over the experimental period (1–35 d), FI was lower in AgNP15 relative to the controls (*p* = 0.004), showing a linear trend (*p* < 0.001). Birds originating from AgNP-injected eggs showed an improved FCR relative to the control, with the lowest FCR exhibited in AgNP15 (*p* < 0.001) and a linear response detected (*p* < 0.001).

### 3.2. Blood Hematological and Biochemical Parameters

The effects of *in ovo* AgNP injection on blood hematological and biochemical parameters of broiler chickens are summarized in Table 3. The rectal temperature was reduced in birds originating from eggs injected with 15 and 20 ppm of AgNPs relative to the NC (*p* = 0.009), showing a linear decrease with increasing AgNP levels (*p* < 0.001). Levels of Hb and PCV increased in birds originating from AgNP-injected eggs versus the controls (*p* < 0.001), with both parameters showing linear (*p* < 0.001) and quadratic responses (*p* = 0.003 and 0.002, respectively).

Serum glucose concentration did not change among treatments according to ANOVA (*p* > 0.05); however, a linear response was observed (*p* = 0.047). The GH level was elevated in AgNP-administered groups versus the controls (*p* < 0.001), with both linear and quadratic changes detected (*p* < 0.001 and *p* = 0.031, respectively). Serum TP, albumin, and globulin levels were higher in birds originating from AgNP-injected eggs than in the controls (*p* < 0.001), with linear and quadratic responses observed (*p* < 0.001–0.031).

Serum ALT activity was higher in AgNP15 and AgNP20 relative to the controls (*p* = 0.001), showing a linear response (*p* < 0.001). On the other hand, AST activity did not differ among groups according to ANOVA (*p* > 0.05), although it showed a linear trend (*p* = 0.017). Serum creatinine levels increased in the AgNP20 group versus the controls (*p* < 0.001), exhibiting a linear response (*p* < 0.001); however, uric acid levels did not differ among groups according to ANOVA (*p* > 0.05), despite a linear trend being detected (*p* = 0.048).

### 3.3. Lipid Profile

The effects of *in ovo* AgNP injection on the serum lipid profile of broiler chickens are summarized in Table 4. Serum TG and LDL-C concentrations were decreased in chickens originating from AgNP-injected eggs relative to the controls (*p* < 0.001). Linear decreases were observed for both TG and LDL-C (*p* < 0.001), while TG also exhibited a quadratic response (*p* = 0.040). The serum HDL-C level was higher in AgNP-administered groups relative to the controls (*p* < 0.001), with both linear (*p* < 0.001) and quadratic (*p* = 0.019) effects reported. No changes among the groups were detected in TC and VLDL-C levels (*p* > 0.05).

### 3.4. Antioxidant and Immune Status

The effects of *in ovo* AgNP injection on the antioxidant and immune status of broiler chickens are presented in Table 5. *In ovo* injection of AgNPs enhanced serum TCA, CAT, and SOD activities, as well as hepatic HSP70 levels compared to the controls (*p* < 0.001). All antioxidant parameters exhibited linear and quadratic responses to increasing levels of AgNPs (*p* < 0.001). The serum MDA level decreased in AgNP-injected groups relative to the controls (*p* < 0.001), showing a linear trend (*p* < 0.001). Serum lysozyme activities and levels of IL1β, INF-γ, IL10, and C3 were elevated in all AgNP-injected groups relative to the controls (*p* < 0.001), with the highest values generally recorded in the AgNP15 group (*p* < 0.001). All immune status-related indices showed linear (*p* < 0.001) and quadratic responses (*p* < 0.001–0.025).

## 4. Discussion

In the present study, we set out to investigate whether the *in ovo* injection of AgNPs could enhance hatchability and post-hatch performance of broiler chickens in a dose-dependent manner, with the objective of recognizing an optimal dosage that improves physiological status without negative impacts. Indeed, hatchability is a primary criterion for evaluating the efficacy of *in ovo* injection in poultry [8,50]. In our study, *in ovo* injection of AgNPs into the developing broiler chickens’ embryos resulted in a significant increase in the hatchability percentage with parallel decreases in embryonic mortalities relative to the control groups, exhibiting both linear and quadratic responses. These findings disagree with earlier reports in which *in ovo* AgNP injection at a dose of 50 µg, either alone or combined with micronutrients such as vitamins, trace minerals, or amino acids, did not affect hatchability percentages or post-hatch growth performance [51]. Similarly, Ahmed et al. [25] reported that neither hatchability percentage nor live chick weights were affected by the *in ovo* injection of AgNPs, probiotics, and their combination at a level of 0.2 mL/egg. Moreover, Pineda et al. [52] recorded no significant changes in hatchability percentage or hatch weight following *in ovo* injection of AgNPs at doses of 10 or 20 mg/kg. The enhancement observed in our study may be owed to the documented beneficial properties of AgNPs in terms of potent antibacterial and immunomodulatory capabilities. Prior reports suggest that AgNPs can influence microbial populations and early immune responses, which may support embryonic development [16,17]. However, it is important to note that antimicrobial competencies and the intestinal microbiota composition were not evaluated in the current study; thus, this mechanism remains untested. Importantly, the enhanced antioxidant status detected in the current study likely contributed to enhanced embryonic resilience against oxidative stress during the late incubation period. This developmental stage is a critical period that is characterized by rapid embryonic growth, elevated metabolic activity, and increased production of reactive oxygen species (ROS), leading to the generation of oxidative stress [53,54,55].

Current findings indicate that AgNPs exerted sustained benefits beyond the embryonic stage, evidenced by enhanced growth performance post-hatch, as demonstrated by improved BW, BWG, and FCR. In contrast, Pineda et al. [52] found that *in ovo* injection of AgNPs followed by continued provision in the drinking water post-hatch negatively affected the growth performance of broiler chickens. This discrepancy may be attributed to differences in exposure duration and dosage. In contrast to the present work, which utilized a singular *in ovo* delivery, Pineda et al. [52] incorporated both *in ovo* and extended post-hatch exposure. Such a combined approach may lead to elevated cumulative nanoparticle levels which impose a metabolic burden and impair growth performance [56,57]. Similarly, Ahmadi and Branch [58] reported increased oxidative stress in broiler chickens supplemented with 20, 40, and 60 ppm of AgNPs in their diets from 1 to 42 d of age, as reflected by increased serum MDA concentrations, with the highest levels observed at 40 and 60 ppm. These findings likely indicate that excessive or prolonged AgNP exposure might impose oxidative imbalance, which could compromise growth performance. In such a context, it is noteworthy that the current study reported both linear and quadratic responses, thereby reinforcing a dose-dependent effect. Particularly, a moderate level (15 ppm) enhanced hatchability and growth performance, suggesting the existence of an ideal biological threshold beyond which further advantages are limited. Meanwhile, the reduction in FI in the AgNP15 group, which was accompanied by an improvement in FCR, may indicate enhanced feed efficiency. This enhanced efficiency may be attributed to reduced metabolic and oxidative burden, which in turn promotes more efficient nutrient utilization and growth [56,57].

The present work attempted to elucidate the physiological and metabolic processes underlying the recorded improvements in post-hatch growth performance. The findings reveal that *in ovo* AgNP administration modulated hematological and biochemical parameters, suggesting an influence on systemic homeostasis in broiler chickens. Notably, RT measured after a 1 h of hyperthermic exposure was lower in birds originating from AgNP-injected eggs, indicating improved thermotolerance and stress resilience in broiler chickens. This response may suggest a possible regulatory effect of the AgNPs on metabolic and hormonal pathways involved in thermal balance and overall health status. To further interpret this response, we analyzed hematological and metabolic indicators. The observed increases in Hb concentration and PCV suggest enhanced oxygen-carrying capacity and improved systemic functionality. Better oxygen transportation may facilitate cellular mechanisms and energy production, thus improving physiological response and maintaining the health status of broiler chickens [59,60]. Supporting this interpretation, earlier research has shown that colloidal AgNPs can stimulate erythropoiesis in chickens exposed to aflatoxins, restoring Hb contents and hematocrit values that were otherwise decreased by toxin-induced hematological damage [61]. Currently, serum glucose concentration, the principal circulating carbohydrate in avian blood [62], did not change among treatments according to ANOVA; however, a linear trend was reported with increasing AgNP levels. This pattern may indicate modulation of carbohydrate metabolism in broiler chickens in response to *in ovo* AgNP administration. In contrast, serum GH concentrations were elevated in all AgNP-administered groups. Growth hormone, a key component of the somatotropic axis, plays a central role in avian growth by stimulating protein synthesis and cell growth [63,64]. Thereby, the increased GH level observed in the current study may partly provide an explanation for the improvement in growth performance of broiler chickens originating from AgNP-injected eggs. Furthermore, the detected increases in TP, albumin, and globulin concentrations in the current study further indicate the enhanced protein synthesis and improved metabolic status [65].

Interestingly, *in ovo* administration of AgNPs at levels of 15 and/or 20 ppm resulted in higher serum ALT activity and creatinine levels, whereas AST activity and uric acid levels did not differ among groups according to ANOVA despite showing linear trends. It is known that the activity of liver enzymes usually increases when they are released from hepatocytes into the blood due to liver dysfunction caused by toxins or viruses [66]. Earlier research by Al-Sultan et al. [67] reported that dietary supplementation with AgNPs at levels of 5–20 mg/kg induced mild to moderate pathologic lesions in the liver and kidneys of broiler chickens. Similarly, histopathological alterations, including mild necrosis and inflammatory cell infiltration in hepatic and renal tissues, have been recorded in response to administration of high doses of AgNPs in broiler chickens [68,69]. Several researchers argued that injurious effects of AgNPs arise from their capacity to produce ROS in the body [70,71]. However, the increases in ALT and creatinine observed in the present study occurred alongside improved growth performance and elevated serum protein fractions (TP, albumin, and globulin), suggesting that the administered doses via *in ovo* injection did not induce overt systemic dysfunction during the post-hatch period. Nevertheless, the dose-related elevations in these biomarkers highlight the need for further investigation, including histopathological assessment of broiler tissues, to confirm long-term safety due to *in ovo* administration.

The results of the current experiment show that *in ovo* administration of AgNPs had a positive effect on the serum lipid profile of broiler chickens, causing decreases in TG and LDL-C concentrations, with an increase in HDL-C levels. Similarly, Ahmed et al. [25] found that *in ovo* injection of AgNPs of 0.2 mL led to a hypolipidemic effect that was indicated by decreases in TC and TG levels in the blood of one-day-old, hatched chicks. In contrast, dietary supplementation of AgNPs for 42 d was associated with elevated TC and TG levels in broiler chickens, indicating a detrimental effect on lipid metabolism [58]. These discrepancies likely reflect differences in exposure route, dosage, and duration, with prolonged post-hatch administration imposing metabolic stress. The decrease in LDL-C, along with a rise in HDL-C, recorded in the current study, indicates enhanced lipid clearance and improved cholesterol transport [72]. HDL-C eliminates surplus cholesterol from the bloodstream and tissues, facilitating its return to the liver for excretion or recycling, whereas LDL-C conveys cholesterol from the liver to peripheral tissues and is more susceptible to oxidative modification [72,73]. Thus, shifting toward higher HDL-C and lower LDL-C is generally indicative for an improved lipid profile. Moreover, the decreased TG level in serum may be attributed to enhanced peripheral lipid utilization [74]. The absence of significant changes in TC and VLDL-C may suggest that overall cholesterol synthesis and hepatic lipid transport were not adversely affected by AgNP *in ovo* administration. Altogether, these findings indicate that *in ovo* administration of AgNPs may improve lipid metabolism and transport in broiler chickens, which could contribute to improved growth performance and overall health status.

In the current study, an enhancement in the antioxidant status was observed, as evidenced by increased activities of TAC, CAT, and SOD, along with a reduced MDA level. These findings indicate that AgNPs improved endogenous antioxidant defenses while attenuating lipid peroxidation. The antioxidant capacity of an organism is a crucial indicator of metabolic stability and immune system balance and is directly linked to overall health status. In contrast, previous studies have reported different findings in response to AgNP exposure under different conditions. For instance, Ognik et al. [75] reported decreases in SOD and CAT activities, as well as an increase in MDA level, in chickens fed diets containing AgNPs for 42 d at a dose of 5 mg/kg body weight. Similarly, Ahmadi and Branch [58] found increased MDA concentrations in chickens offered dietary AgNPs at doses of 20, 40, and 60 ppm/kg for 42 d. These earlier findings have been attributed to excessive or prolonged nanoparticle exposure, which can get into the mitochondria, resulting in lipid peroxidation and subsequent damage to mitochondrial proteins and nucleic acids and generation of ROS. In response, cells activate antioxidant enzymes, including SOD, glutathione peroxidase, and CAT, to counteract ROS-induced damage [76,77]. However, when oxidative stress exceeds the capacity of these defenses, depletion of antioxidant enzymes may occur, resulting in reduced enzymatic activities, as previously observed in chickens exposed to dietary AgNPs for prolonged periods. In contrast, the enhanced antioxidant enzyme activities observed in the current study may suggest that the *in ovo* doses administered did not surpass cellular defenses but instead triggered adaptive protective mechanisms.

The increase in hepatic HSP70 further supports better cellular stress response. HSPs are usually maintained at a low level in the body under normal physiological conditions. However, in response to sudden environmental temperature changes, HSPs are expressed to support the body’s resilience against stress-induced damage [78,79]. HSPs serve as protective proteins through binding and stabilizing cytoskeletal proteins when exposed to denaturation caused by ROS [80]. HSP70 is the most frequently researched member of the HSP family, owing to its possible protective attributes against various stresses [81]. The mutual enhancement in antioxidant defenses and HSP70 levels in this study suggest that AgNP administration may facilitate a coordinated cellular protection mechanism encompassing both redox control and stress protein response.

In addition to its antioxidant effect, *in ovo* AgNP administration modulated the immune status in broiler chickens, as indicated by increased serum lysozyme activity and elevated concentrations of C3 and cytokines IL-1β, IFN-γ, and IL10. C3 plays a central role in modulating immune status by initiating complement activation cascades that induce inflammation as a defensive response to foreign invaders; however, lysozyme, a naturally occurring antimicrobial enzyme, represents a cornerstone of innate immunity [82,83]. Lysozyme’s antimicrobial activity is achieved by hydrolyzing β–1,4-glycosidic bonds between N-acetylmuramic acid and N-acetyl glucosamine, which are key components of peptidoglycans in bacterial cell walls [82,84]. Furthermore, lysozymes can regulate immune response through induction of immunoglobulin (Ig) A secretion through the hydrolysis of products like muramyl dipeptide and may support gut barrier function, which in turn could contribute to improved growth performance [85,86,87]. The recorded elevations in IL-1β, IFN-γ, and IL10 further signify the regulation of both pro-inflammatory and anti-inflammatory pathways. IFN-γ has been recognized as a crucial pro-inflammatory cytokine that is involved in the activation of cellular immunity and regulation of the host defense mechanism, while IL1β is significant in regulating the acute phase response to stress during stressful situations [88,89,90]. Conversely, IL10 acts as a key anti-inflammatory cytokine that regulates excessive immune activation and preserves immunological homeostasis [91,92]. The simultaneous increase in both pro- and anti-inflammatory cytokines may indicate a balanced immunomodulatory response instead of uncontrolled inflammation; however, further investigation is necessary to elucidate the mechanisms underlying cytokine modulation in response to AgNP exposure. Earlier research has reported variable effects of AgNP exposure on immune responses. For instance, Bhanja et al. [93] demonstrated that *in ovo* injection of 15 mg of AgNPs per egg led to downregulation of hepatic pro-inflammatory cytokines IL6 and IL12 in chicken embryos. Moreover, Pineda and colleagues reported inconsistent effects on humoral immunity, ranging from no changes in IgG and IgM levels following combined pre- and post-hatch exposure to decreased IgG concentration when AgNPs were administered only during the post-hatch period [52,94]. These findings may suggest that AgNPs may differentially influence immune pathways depending on exposure timing and duration. In this study, humoral immune indicators were not assessed; therefore, further investigation is required to determine the effects of *in ovo* AgNP administration on antibody-mediated immunity.

## 5. Conclusions

Based on the present findings, it can be concluded that *in ovo* administration of AgNP particles has the potential to enhance hatchability and reduce embryonic mortality in broiler chickens. The administration of *in ovo* AgNPs improved growth performance and physiological status post-hatch, as indicated by positive modulation of the assessed hematological and biochemical indices. *In ovo* AgNP administration also improved the lipid profile, antioxidant status, and immune-related responses post-hatch. The observed effects were dose-dependent, with the moderate level (15 ppm) generally showing better responses. Hence, *in ovo* administration of appropriate levels of AgNPs may represent a potential strategy for enhancing embryonic viability and subsequent performance in broiler chickens. While our findings support the short-term advantages of *in ovo* AgNP administration at moderate levels, additional research is necessary to assess tissue distribution and environmental effects, particularly in light of the observed changes in liver enzyme activity and kidney function markers.

## Figures and Tables

**Table 1:** Ingredient composition and nutrient content [%] of basal diet [as-fed basis].

Item	Starter (0–12 d)	Grower 1 (13–28 d)	Grower 2 (29–35 d)
Yellow corn	52.30	54.50	60.32
Soybean meal (46% CP)	32.32	29.00	26.95
Corn gluten (60% CP)	3.48	1.65	1.85
Soy oil	2.21	2.96	3.28
Wheat middling	4.89	7.67	3.51
Limestone	0.85	0.85	0.89
Dicalcium phosphate	2.40	1.80	1.65
Common salt	0.18	0.19	0.19
Sodium bicarbonate	0.41	0.41	0.41
Premix *	0.30	0.30	0.30
DL-Methionine, 98%	0.15	0.18	0.16
Lysine, HCl, 78%	0.25	0.23	0.24
Phytase	0.02	0.02	0.02
Choline chloride	0.24	0.24	0.24
Calculated nutrient levels			
Metabolizable energy, kcal/kg	2900	2950.22	3050.24
Crude protein %	22.04	20.00	19.00
Ether extract %	4.69	5.53	5.94
Crude fiber %	3.82	3.82	3.50
Calcium %	0.96	0.82	0.80
Available phosphorus %	0.58	0.47	0.43
Lysine %	1.26	1.16	1.10
Methionine %	0.48	0.47	0.44

* Vitamin and mineral premix per kg of diet: vitamin A, 10,000 IU; vitamin D3, 5000 IU; vitamin E, 80 IU; vitamin K, 3 g; thiamine, 3 g; riboflavin, 9 g; pantothenic acid, 15 g; folic acid, 2 g; pyridoxine, 4 g; niacin, 60 g; cobalamin, 20 µg; biotin, 0.15 mg; Fe, 40 mg; Cu, 15 mg; Mn, 100 mg; Zn, 100 mg; I, 1 mg; Se, 0.35 g.

**Table 2:** Effects of *in ovo* silver nanoparticle administration on hatchability, body weight at hatch, and growth performance of broiler chickens.

	*In ovo* Silver Nanoparticles, ppm							*p*-Value		
	0 (NC)	0 (VC)	AgNP10	AgNP15	AgNP20	SEM	F-Statistic	ANOVA	Linear	Quadratic
Hatchability (% of set eggs)	80.83 ^bc^	80.66 ^c^	85.00 ^a^	86.16 ^a^	84.00 ^ab^	0.537	9.15	<0.001	<0.001	0.036
Hatchability (% of injected eggs at d 18)	84.50 ^b^	83.66 ^b^	88.16 ^a^	87.83 ^a^	87.33 ^a^	0.412	14.34	<0.001	<0.001	0.110
Embryonic mortality (% post-injection)	15.50 ^a^	16.33 ^a^	11.83 ^b^	12.16 ^b^	12.66 ^b^	0.412	13.97	<0.001	<0.001	0.055
BW at hatch, g	40.83 ^bc^	40.66 ^c^	42.00 ^ab^	42.33 ^a^	41.83 ^abc^	0.177	5.72	0.002	<0.001	0.162
Initial BW, g	41.16	41.50	41.33	41.50	41.33	0.089	0.449	0.772	0.617	0.400
BW at 35 d, g	1831.50 ^c^	1813.66 ^c^	1937.16 ^b^	2136.66 ^a^	2009.66 ^b^	24.15	32.99	<0.001	<0.001	0.114
BWG (1–35 d), g	1790.33 ^c^	1772.16 ^c^	1895.83 ^b^	2095.16 ^a^	1968.33 ^b^	24.14	32.99	<0.001	<0.001	0.115
FI (1–35 d), g	2984.16 ^a^	2985.83 ^a^	2912.66 ^ab^	2896.66 ^c^	2904.83 ^ab^	10.95	5.14	0.004	<0.001	0.347
FCR (1–35 d)	1.66 ^a^	1.68 ^a^	1.53 ^b^	1.38 ^c^	1.47 ^bc^	0.023	34.23	<0.001	<0.001	0.089

^a,b,c^ Means with different superscripts within the same row differ significantly (*p* < 0.05). NC: non-injected control; VC: vehicle control; AgNP10: AgNPs at 10 ppm; AgNP15: AgNPs at 15 ppm; AgNP20: AgNPs at 20 ppm; SEM: standard error of means; BW: body weight; BWG: body weight gain; FI: feed intake; FCR: feed conversion ratio.

**Table 3:** Effects of *in ovo* silver nanoparticle administration on physiological status and blood biochemical parameters of broiler chickens.

	*In ovo* Silver Nanoparticles, ppm							*p*-Value		
	0 (NC)	0 (VC)	AgNP10	AgNP15	AgNP20	SEM	F-Statistic	ANOVA	Linear	Quadratic
BT °C	40.80 ^a^	40.14 ^ab^	39.83 ^ab^	39.66 ^b^	39.76 ^b^	0.128	4.23	0.009	<0.001	0.133
Hb, g/dL	10.96 ^b^	10.99 ^b^	12.83 ^a^	12.88 ^a^	12.92 ^a^	0.193	24.57	<0.001	<0.001	0.033
PCV, %	34.20 ^b^	34.58 ^b^	35.67 ^a^	35.93 ^a^	35.70 ^a^	0.144	23.11	<0.001	<0.001	0.002
Glucose, mmol/L	340.16	340.66	341.33	341.00	341.50	0.208	1.38	0.268	0.047	0.562
GH, ng/mL	2.23 ^b^	2.23 ^b^	3.58 ^a^	3.75 ^a^	3.68 ^a^	0.146	26.33	<0.001	<0.001	0.031
TP, g/dL	3.40 ^d^	3.41 ^d^	4.54 ^c^	4.82 ^a^	4.67 ^b^	0.116	793.05	<0.001	<0.001	<0.001
Albumin, g/dL	1.47 ^b^	1.46 ^b^	2.26 ^a^	2.35 ^a^	2.41 ^a^	0.084	58.59	<0.001	<0.001	0.024
Globulin, g/dL	1.92 ^b^	1.95 ^b^	2.28 ^a^	2.46 ^a^	2.25 ^a^	0.046	12.91	<0.001	<0.001	0.019
ALT, U/L	6.33 ^b^	6.16 ^b^	7.16 ^ab^	8.16 ^a^	7.83 ^a^	0.207	6.29	0.001	<0.001	0.803
AST, U/L	44.66	43.83	47.33	47.83	47.66	0.598	2.31	0.086	0.017	0.721
Creatinine, mg/dL	0.221 ^c^	0.231 ^bc^	0.241 ^ab^	0.248 ^ab^	0.251 ^a^	0.002	8.10	<0.001	<0.001	0.314
Uric acid, mg/dL	1.95	1.95	1.99	1.99	1.98	0.007	1.65	0.192	0.048	0.462

^a,b,c,d^ Means with different superscripts within the same row differ significantly (*p* < 0.05). NC: non-injected control; VC: vehicle control; AgNP10: AgNPs at 10 ppm; AgNP15: AgNPs at 15 ppm; AgNP20: AgNPs at 20 ppm; SEM: standard error of means; BT: rectal temperature; Hb: hemoglobin; PCV: packed cell volume; GH; growth hormone; TP: total protein; ALT: alanine aminotransferase; AST: aspartate aminotransferase.

**Table 4:** Effects of *in ovo* silver nanoparticle administration on lipid profile of broiler chickens.

	*In ovo* Silver Nanoparticles, ppm							*p*-Value		
	0 (NC)	0 (VC)	AgNP10	AgNP15	AgNP20	SEM	F-Statistic	ANOVA	Linear	Quadratic
TC, mg/dL	3.40	3.44	3.31	3.29	3.33	0.023	1.68	0.184	0.067	0.533
TG, mg/dL	1.28 ^a^	1.30 ^a^	1.13 ^b^	1.11 ^b^	1.12 ^b^	0.017	28.83	<0.001	<0.001	0.040
HDL-C, mg/dL	1.98 ^b^	1.99 ^b^	2.20 ^a^	2.22 ^a^	2.21 ^a^	0.022	27.87	<0.001	<0.001	0.019
LDL-C, mg/dL	1.17 ^a^	1.21 ^a^	0.881 ^b^	0.833 ^b^	0.890 ^b^	0.040	8.19	<0.001	<0.001	0.183
VLDL-C, mg/dL	0.240	0.233	0.225	0.228	0.223	0.002	1.05	0.398	0.077	0.548

^a,b^ Means with different superscripts within the same row differ significantly (*p* < 0.05). NC: non-injected control; VC: vehicle control; AgNP10: AgNPs at 10 ppm; AgNP15: AgNPs at 15 ppm; AgNP20: AgNPs at 20 ppm; SEM: standard error of means; TC: total cholesterol; TG: total triglycerides; HDL-C: high-density lipoprotein cholesterol; LDL-C: low-density lipoprotein cholesterol; VLDL-C: very low-density lipoprotein cholesterol.

**Table 5:** Effects of *in ovo* silver nanoparticle administration on antioxidant and immune status of broiler chickens.

	*In ovo* Silver Nanoparticles, ppm							*p*-Value		
	0 (NC)	0 (VC)	AgNP10	AgNP15	AgNP20	SEM	F-Statistic	ANOVA	Linear	Quadratic
TAC, U/mL	10.04 ^c^	10.08 ^c^	13.06 ^b^	13.24 ^a^	13.17 ^a^	0.282	4317.83	<0.001	<0.001	<0.001
CAT, U/mL	2.33 ^b^	2.24 ^b^	4.15 ^a^	4.30 ^a^	4.24 ^a^	0.178	724.27	<0.001	<0.001	<0.001
SOD, U/mL	131.35 ^b^	131.61 ^b^	147.22 ^a^	147.46 ^a^	147.42 ^a^	1.45	770.69	<0.001	<0.001	<0.001
MDA, nmol/mL	5.05 ^a^	5.16 ^a^	2.57 ^b^	2.16 ^b^	2.21 ^b^	0.285	25.89	<0.001	<0.001	0.077
HSP70, ng/mg	2.32 ^b^	2.34 ^b^	3.98 ^a^	4.12 ^a^	4.07 ^a^	0.164	68.68	<0.001	<0.001	<0.001
Lysozyme, µg/mL	131.00 ^c^	132.50 ^c^	149.33 ^b^	158.33 ^a^	150.16 ^b^	2.11	47.41	<0.001	<0.001	<0.001
IL-1β, µg/mL	141.83 ^c^	141.50 ^c^	152.00 ^b^	162.00 ^a^	156.00 ^b^	1.58	45.50	<0.001	<0.001	0.025
IFN-γ, pg/mL	6.93 ^b^	7.01 ^b^	10.71 ^a^	11.15 ^a^	11.08 ^a^	0.373	152.35	<0.001	<0.001	<0.001
IL10, pg/mL	1.41 ^b^	1.45 ^b^	2.85 ^a^	3.21 ^a^	3.11 ^a^	0.160	42.12	<0.001	<0.001	0.019
C3, g/L	1.09 ^b^	1.08 ^b^	1.23 ^a^	1.27 ^a^	1.25 ^a^	0.015	65.67	<0.001	<0.001	0.009

^a,b,c^ Means with different superscripts within the same row differ significantly (*p* < 0.05). NC: non-injected control; VC: vehicle control; AgNP10: AgNPs at 10 ppm; AgNP15: AgNPs at 15 ppm; AgNP20: AgNPs at 20 ppm; SEM: standard error of means; TAC: total antioxidant capacity; CAT: catalase enzyme; SOD: superoxide dismutase enzyme; MDA: malondialdehyde; HSP70: heat shock protein 70; IL1β: interleukin1β; IFN-γ: interferon-γ: IL10: interleukin10; C3: complement 3.

## Data Availability

The original contributions presented in the study are included in the article; further inquiries can be directed to the corresponding author.
